# A structured music-based intervention for motor rehabilitation and exploratory cognitive and quality-of-life outcomes after stroke: a study protocol for a randomized waitlist-controlled intervention study

**DOI:** 10.3389/fneur.2026.1822193

**Published:** 2026-07-01

**Authors:** Aída Estévez, Miguel Ángel Pérez Nieto, Laura Herrero

**Affiliations:** 1 Faculty of Health Sciences - HM Hospitals, University Camilo José Cela, Madrid, Spain; 2 HM Hospitals Health Research Institute, Madrid, Spain

**Keywords:** executive functions, motor functions, motor rehabilitation, music-based intervention, quality of life, stroke, waitlist-controlled intervention

## Abstract

**Background:**

Stroke is one of the leading causes of long-term disability and is frequently associated with persistent motor, cognitive, emotional, and social sequelae that affect autonomy and quality of life. Music-based interventions have shown potential benefits in post-stroke rehabilitation, particularly through rhythmic auditory stimulation, structured instrumental practice, and active musical engagement. These approaches may support motor coordination and motivation and may also contribute to emotional regulation, social interaction, and perceived quality of life. However, evidence remains limited regarding structured music-based interventions that integrate motor activities with broader cognitive and psychosocial components into post-stroke rehabilitation. This randomized waitlist-controlled intervention study aims to evaluate the effects of a structured music-based intervention on motor function after stroke, with executive functioning and stroke-specific quality of life being examined as exploratory outcomes.

**Methods:**

A prospective, phased, randomized waitlist-controlled intervention study will include adults aged 18–70 years who have experienced a stroke at least 3 months before enrollment. The first recruitment phase will be conducted in a single outpatient neurological rehabilitation center in the Community of Madrid, Spain, with subsequent recruitment phases planned at additional outpatient neurological rehabilitation centers within the Community of Madrid until the required sample size is achieved. Two groups will be formed: an experimental group receiving standard rehabilitation care plus a structured music-based intervention consisting of 10 sessions and a waitlist group receiving only standard rehabilitation care during this period, followed by the structured music-based intervention. Assessments will be carried out at week 0 (baseline), week 5 (post-intervention), and week 10 (after group crossover), with an additional follow-up at 3 months. The primary outcome will be motor function. Upper-limb motor function will be assessed as a secondary motor outcome, while executive functioning and stroke-specific quality of life will be examined as exploratory outcomes. Analyses will follow the intention-to-treat principle, with additional analyses conducted for participants completing at least 80% of sessions. A linear mixed-effects model will be used to estimate the main effect of the intervention on the primary motor outcome, with additional secondary and exploratory models performed for upper-limb motor function, executive functioning, and stroke-specific quality of life. The model will include time, group, and the interaction between group and time as fixed effects, with time since stroke included as a covariate. Additional analyses will be conducted using intraclass correlation coefficients to explore the agreement between participant and family member reports of executive function.

**Discussion:**

This protocol will evaluate a structured music-based intervention as a complement to standard rehabilitation care, with a primary focus on motor rehabilitation. Exploratory analyses will examine whether the intervention is associated with changes in executive functioning and stroke-specific quality of life. The study is expected to provide preliminary evidence on the feasibility and potential effects of this integrative music-based intervention within post-stroke rehabilitation and to contribute to the development of multidomain rehabilitation approaches. The findings may inform the design of future studies and the potential implementation of music-based interventions in post-stroke rehabilitation settings.

**Study protocol preregistration:**

https://osf.io/hqruw.

## Introduction

Stroke is one of the leading causes of long-term disability and comprises ischemic and hemorrhagic subtypes (1). In addition to the acute phase, stroke survivors often experience persistent sequelae that compromise autonomy, participation, and quality of life (2, 3). Motor impairments, executive dysfunction, and deterioration of emotional and social wellbeing are among the most relevant consequences of stroke. These areas represent fundamental aspects of post-stroke rehabilitation and therefore constitute the core focus of this protocol (4).

Post-stroke neurorehabilitation is increasingly understood as a multidimensional and multidisciplinary process that should address not only body functions but also activity, participation, and quality of life (4, 5). However, standard rehabilitation approaches often address motor, cognitive, and emotional domains separately, which may limit their ability to capture the dynamic interaction between these domains in everyday functioning. Cognitive impairments may reduce engagement in motor training, while emotional disturbances can exacerbate functional limitations and negatively affect recovery trajectories (4, 6, 7). Integrative rehabilitation approaches may therefore provide added value by promoting coordinated stimulation across domains and by increasing the functional relevance of rehabilitation tasks.

Within this context, music-based interventions represent a potentially valuable complementary approach in post-stroke rehabilitation due to their multisensory, rhythmic, motivational, and socially engaging nature. Over the past two decades, multiple studies have explored music-based interventions in post-stroke rehabilitation, primarily focusing on isolated domains such as motor recovery, cognitive function, or emotional wellbeing. Evidence from randomized controlled trials has shown that rhythmic auditory stimulation can enhance gait and upper-limb coordination, while structured instrumental practice supports fine motor skills and functional recovery mechanisms (8, 9). Other studies have emphasized the cognitive and emotional benefits of music-based interventions, highlighting improvements in executive functions, attention, and mood regulation (10, 11). Moreover, systematic reviews have indicated that participation in active musical activities contributes to social engagement and perceived quality of life by reducing symptoms of depression and anxiety and promoting self-efficacy and motivation during rehabilitation (12–14).

Despite this growing body of evidence, most existing interventions target these domains separately, focusing either on motor outcomes or on emotional and cognitive aspects, without fully integrating them into a single, comprehensive rehabilitation model. Although multidomain rehabilitation models are beginning to emerge in stroke care, structured music-based intervention protocols specifically designed to integrate motor rehabilitation with exploratory executive and quality-of-life-related components within a single coherent intervention remain limited (11, 14, 15). The present study seeks to address this gap by evaluating a structured music-based intervention primarily focused on motor rehabilitation while also exploring potential changes in executive functioning and quality of life as broader rehabilitation-related outcomes.

From a motor perspective, participants frequently present with hemiparesis, loss of coordination, and limitations in fine motor skills, which interfere with activities of daily living. Despite standard rehabilitation care, motor recovery is often incomplete, prompting the search for complementary strategies to support mechanisms associated with motor relearning and functional recovery (2). Evidence suggests that music, particularly through rhythmic auditory stimulation and structured musical engagement, can support motor and cognitive recovery processes (8, 9, 14). In addition, rhythmic auditory stimulation has been shown to improve gait synchronization (16), while instrumental practice may enhance bimanual coordination and fine motor function (17). Clinical trials have demonstrated that exercises using simple instruments or electronic keyboards improve manual dexterity and motor precision, in addition to boosting motivation and adherence to treatment (8, 13, 18). A recent systematic review has reported beneficial effects of music therapy interventions on hand function in patients with stroke (9). These findings support the inclusion of rhythmic and instrumental tasks in this protocol as a complementary strategy to support motor rehabilitation.

With regard to executive functions, many participants exhibit deficits in attention, working memory, planning, and cognitive flexibility, which have a direct impact on behavioral organization and adaptation to daily life (6, 7, 11). Clinical observations and case-based reports further illustrate the presence of these impairments in post-stroke populations (19). Recent studies have suggested that active music practice may engage networks involved in auditory–motor integration and cognitive control (10, 20), facilitating attentional regulation, inhibition of automatic responses, and the ability to switch strategies. Structured musical activities, such as following rhythmic patterns, improvising, or coordinating musical sequences, have been used to target executive processes, with emerging evidence of cognitive benefits (21). A recent review has also highlighted the potential of music-based interventions to improve executive function and emotional wellbeing after stroke (11). The proposed program incorporates structured musical activities requiring planning, working memory, and cognitive flexibility, with the aim of increasing cognitive engagement during motor and musical tasks.

Post-stroke quality of life is affected not only by physical and cognitive limitations but also by the high prevalence of emotional disorders such as depression and anxiety. These conditions significantly reduce social participation and adherence to treatment (3, 19). Research indicates that daily music listening can improve mood and perceived wellbeing (16). Moreover, participation in active group sessions fosters social interaction and reduces feelings of isolation (13). Participation in music-based activities may support social engagement and emotional wellbeing after stroke (13, 14). Some programs have reported improvements in stroke-specific quality-of-life scores, demonstrating that music provides benefits beyond the motor domain (14). This protocol includes a group-based, integrative format designed to harness these positive effects on quality of life and emotional wellbeing.

Taken together, the available evidence suggests that music acts as a multisensory and motivating stimulus capable of influencing motor function (8, 16), cognition (11, 21), and emotional wellbeing (11, 14). Building on this rationale, this protocol proposes a ten-session integrative music-based intervention primarily designed to support motor recovery through rhythmic and instrumental tasks. The program also includes structured activities involving planning, working memory, cognitive flexibility, social interaction, emotional regulation, and motivation, which will be examined as exploratory components in relation to executive functioning and quality of life.

Although structured music-based and music-supported rehabilitation protocols have recently begun to be explored in post-stroke populations, evidence remains limited, particularly regarding integrative approaches addressing multiple rehabilitation-related domains simultaneously (22).

Accordingly, the program is presented as a structured integrative model that builds on previous evidence on music-based interventions, neurologic music therapy, and music-supported rehabilitation after stroke. The primary aim of this study is to evaluate the effects of the intervention on motor function. Executive functioning and stroke-specific quality of life will be examined as exploratory outcomes, given the brief intervention dose and the multifactorial nature of change in these domains.

## Methods and design

### Context of post-stroke rehabilitation and rationale for the intervention

Post-stroke neurorehabilitation is typically based on multidisciplinary approaches involving physiotherapy, occupational therapy, speech and language therapy, and neuropsychological interventions (4, 5). These treatments primarily focus on restoring motor function, improving communication abilities, and addressing cognitive deficits, depending on the participant’s clinical profile.

In recent years, there has been increasing recognition of the need for more comprehensive and participant-centered rehabilitation models that address not only impairments at the body function level but also activity, participation, and quality of life (4, 5). Multidisciplinary and, more recently, integrative approaches aim to combine different therapeutic modalities to enhance functional recovery and promote engagement in meaningful activities (15).

However, despite this shift toward multidimensional rehabilitation, the majority of existing interventions are still delivered in a domain-specific manner, with motor, cognitive, and emotional aspects often addressed separately across different therapeutic disciplines (6, 7). This fragmentation may limit the ability of rehabilitation programs to capture the dynamic interaction between these domains in real-life functioning.

Within this context, the proposed music-based intervention represents a potentially valuable complementary approach due to its inherently multisensory and integrative nature. Musical activities simultaneously engage motor, cognitive, and emotional processes, offering an opportunity to address these domains within a unified rehabilitation-oriented framework (11, 14).

This protocol aims to address this gap by proposing a structured music-based program primarily focused on motor rehabilitation while incorporating executive and quality-of-life-related components as exploratory domains within a single coherent intervention. The program combines rhythmic, instrumental, and structured musical activities to support motor rehabilitation while also engaging cognitive and emotional processes.

### Study design

This is a prospective, phased, randomized waitlist-controlled intervention study with a parallel-group design, aimed primarily at evaluating the effects of the intervention on motor function. Upper-limb motor function will be assessed as a secondary motor outcome, while executive functioning and stroke-specific quality of life will be examined as exploratory outcomes. Participants will be randomly allocated to either an experimental group or a control/waitlist group.

The study will be implemented in successive recruitment phases. The first phase will be conducted in a single outpatient neurological rehabilitation center in the Community of Madrid, Spain, and subsequent phases will extend recruitment to additional outpatient neurological rehabilitation centers within the Community of Madrid until the required total sample size is achieved.

In the first recruitment phase, approximately 24 participants are expected to be included, with 12 participants allocated to each group. Recruitment will then continue in subsequent phases across additional centers, maintaining the same eligibility criteria, intervention protocol, assessment schedule, randomization procedures, and outcome measures, until the planned sample of 76 participants is reached.

Two groups will undergo the same overall rehabilitation process. Over a total of 5 weeks (two sessions per week), the experimental group will receive their standard rehabilitation care complemented by a program of music-based activities. The control/waitlist group will receive only standard rehabilitation care during the first 5-week intervention period and will subsequently undergo the music-based program after the experimental group has completed it, thereby effectively switching their roles.

This design ensures that all participants benefit from the intervention while allowing temporal comparison between the groups. This structure is consistent with previous studies of similar music-based rehabilitation programs and supports ethical rigor by ensuring that all participants receive a potentially beneficial intervention.

All participants will be assessed at baseline (week 0), after 5 weeks (post-intervention), and again at 10 weeks (after crossover). A 3-month follow-up will be conducted to explore the maintenance of potential effects.

The study has been preregistered in OSF Registries.^1^ This record is reported as an open science preregistration and is not presented as a clinical trial registry.

### Recruitment

Participants will be recruited in successive phases from outpatient neurological rehabilitation centers in the Community of Madrid, Spain. The first recruitment phase will take place in a single specialized outpatient neurological rehabilitation center, where patients are typically in the late subacute or chronic phase of recovery (≥3 months post-stroke). Based on the estimated annual recruitment capacity and eligible patient flow at this center, approximately 24 eligible and recruitable participants are expected to be included during the first recruitment phase, with 12 participants allocated to each group.

Subsequent recruitment phases will extend to additional outpatient neurological rehabilitation centers within the Community of Madrid to reach the required total sample size of 76 participants. All participating centers will follow the same eligibility criteria, intervention protocol, assessment schedule, randomization procedures, and data collection methods. Recruitment will continue until the planned total sample size has been achieved, with completion of recruitment expected within approximately 1 year.

This is a non-pharmacological, music-based rehabilitation intervention. It does not involve changes to participants’ usual medical care or prescribed medication. Participants will receive written information about the study along with the informed consent form. Those who voluntarily agree to participate will be randomly assigned to the experimental or control/waitlist group.

Eligibility screening will be conducted by trained rehabilitation professionals using a medical record review, clinical interview, Barthel Index scoring, the Montreal Cognitive Assessment (MoCA), and functional screening of motor and receptive language abilities.

#### Inclusion criteria

The inclusion criteria will include the following:

- Participants must be between 18 and 70 years of age. The upper age limit was established to reduce heterogeneity associated with age-related comorbidities and cognitive decline, which may influence rehabilitation outcomes and engagement in structured interventions. This range is also consistent with previous research on post-stroke rehabilitation and functional recovery mechanisms (6, 7).
- Participants must have a stroke diagnosis made at least 3 months prior to participation, corresponding to the late subacute phase (3–6 months post-stroke) and/or the chronic phase (>6 months), in line with commonly used temporal classifications of post-stroke recovery phases (5). These classifications distinguish subacute and chronic stages based on time since stroke onset, thereby helping to ensure clinical stability and reduce variability associated with earlier stages of recovery.
- Participants must have sufficient cognitive and receptive language abilities to understand and follow simple instructions, regardless of verbal expression capacity. Cognitive screening will be conducted using the Montreal Cognitive Assessment (MoCA), with a score of ≥18 used as the eligibility threshold. Receptive language and task comprehension will also be confirmed during screening through the ability to follow simple one-step verbal instructions.
- Participants must have a minimum functional ability to perform basic activities of daily living, defined as a Barthel Index score of ≥40, together with sufficient sitting tolerance and the ability to engage in structured activities, with support if needed. Participants must have sufficient motor ability to perform the planned intervention tasks, as determined during screening through observation of seated postural control and basic upper-limb performance on intervention-related tasks.
- Participants must not have severe pre-stroke cognitive or functional impairments, based on medical records and clinical history.

#### Exclusion criteria

The exclusion criteria will include the following:

- Participants must be over 70 years old.
- Participants must have other neurological or neurocognitive conditions unrelated to stroke, including pre-existing dementia, traumatic brain injury, neurodegenerative disorders, or documented pre-stroke cognitive decline.
- Participants must be less than 3 months post-stroke.
- Participants must have severe impairments in language comprehension preventing the understanding of simple instructions, severe motor impairments, or complete dependence in activities of daily living.

### Study setting

The first recruitment phase will take place in a specialized outpatient neurological rehabilitation center in the Community of Madrid, Spain, where both intervention sessions and assessments will be conducted. In subsequent phases, recruitment will be extended to additional outpatient neurological rehabilitation centers within the Community of Madrid. All participating centers will use the same intervention protocol, assessment procedures, facilitator training requirements, and fidelity monitoring procedures to ensure consistency across sites.

### Sample size

The sample size was calculated following recommendations for comparing two means in intervention studies (23). As the Fugl-Meyer Assessment (FMA) was the main outcome, change in this measure was selected as the primary criterion. Following previous research (24), the minimal difference in scores used to detect between-group differences in motor improvement was 4, assuming a standard deviation of 12. Using a two-tailed t-test for the difference between means with 80% power and a 5% level of significance, the sample size required was 70 participants, with 35 per group. Considering a dropout rate of 10%, the final required sample size was estimated at 76 participants, with 38 per group.

Given the specificity of the target population and the outpatient rehabilitation context, recruitment will be conducted in successive phases. The first recruitment phase, conducted in a single center, is expected to include approximately 24 eligible and recruitable participants, with 12 participants per group. Subsequent phases will include additional outpatient neurological rehabilitation centers in the Community of Madrid until the total required sample size of 76 participants is reached. Based on the projected recruitment capacity across participating centers, completion of recruitment is expected within approximately 1 year.

### Randomization and allocation

The study follows a randomized waitlist-controlled design:

- Experimental group: standard rehabilitation care plus music-based intervention during the first intervention phase.
- Control/waitlist group: standard rehabilitation care during the first intervention phase, followed by music-based intervention after completion of the experimental group intervention.

Random allocation will take place after obtaining informed consent.

**Figure 1:**
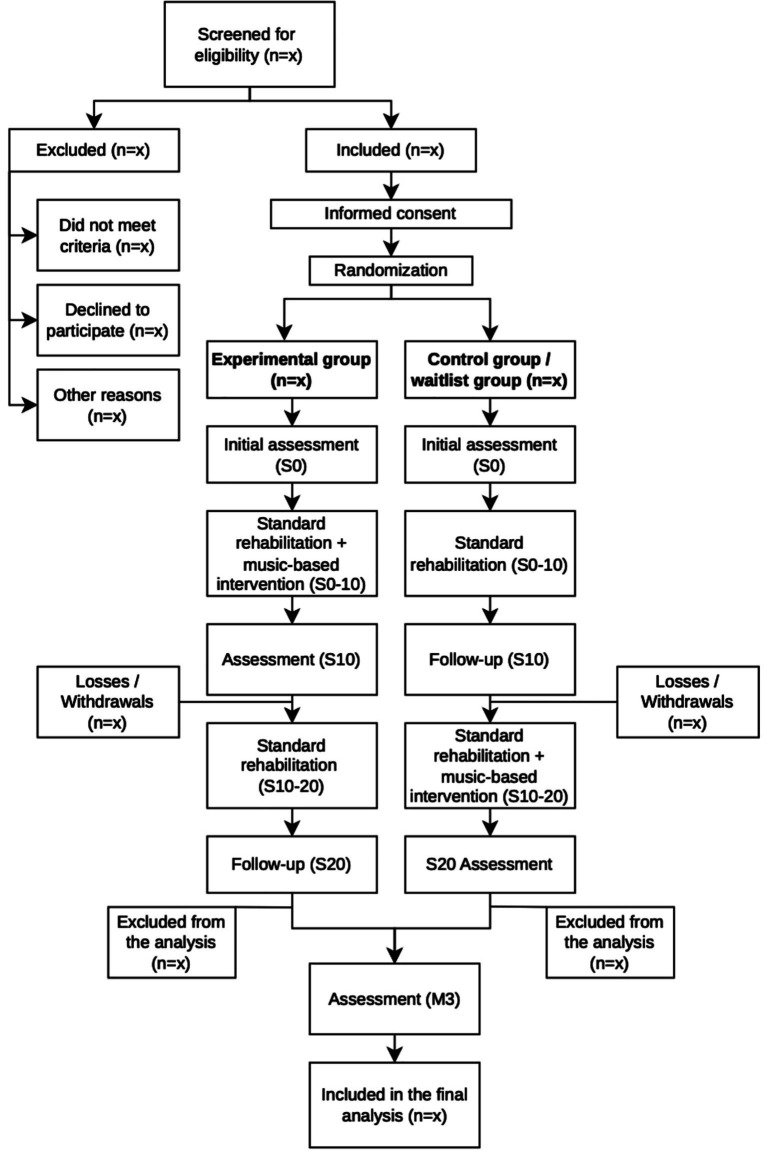
Flow diagram of participant screening, recruitment, randomization, intervention, crossover, and follow-up across recruitment phases.

The selection, randomization, and follow-up process will be represented in a flow diagram (Figure 1), consistent with recommendations for waitlist-controlled trials. Randomization will be generated by a member of the research team using simple randomization software. If recruitment is extended to additional centers, randomization will be monitored across centers to maintain balance between groups within each recruitment phase. Group allocation will be concealed until the point of assignment to ensure masking of the sequence.

### Blinding

Due to the nature of the music-based intervention, neither participants nor the professionals delivering the intervention can be blinded. Outcome assessors will not be blinded due to logistical reasons. To minimize potential bias, standardized assessment procedures will be strictly followed, and assessors will receive prior training in the administration of all outcome measures to ensure consistency in data collection. In addition, objective and widely validated instruments will be used whenever possible.

The statistician will not be involved in intervention delivery or outcome assessment and will be blinded to group allocation during the primary analysis. Groups will be coded as Group A and Group B until the primary analysis has been completed to reduce potential analytical bias.

### Interventions

#### Groups

Two parallel groups will be formed: the experimental group and the control/waitlist group.

#### Standard rehabilitation care

Post-stroke rehabilitation typically addresses a range of sequelae affecting motor function, communication, cognition, and activities of daily living. Common impairments include hemiparesis, coordination deficits, language disturbances, and executive dysfunction, which require multidisciplinary intervention to support functional recovery and independence.

All participants will continue their prescribed standard rehabilitation care. This typically includes a combination of physiotherapy, occupational therapy, speech and language therapy, and, when indicated, neuropsychological intervention, as prescribed in the clinical setting.

In the outpatient rehabilitation settings participating in the study, standard rehabilitation care will be delivered according to each center’s usual clinical practice. In the first recruitment center, patients typically attend the rehabilitation center from Monday to Friday between 9:00 and 16:30 to receive individualized and group therapy sessions. In subsequent centers, the frequency and combination of therapies will be recorded according to each participant’s clinical needs and rehabilitation plan.

During the study period, participants will be required to maintain a stable rehabilitation schedule to minimize variability. To ensure comparability between groups, detailed information on the type of therapy, frequency, and session duration will be systematically recorded for each participant at baseline and monitored throughout the intervention using a standardized log. Any relevant changes in rehabilitation intensity or type of therapy will be documented and considered in the interpretation of the results.

This approach allows the study to reflect real-world clinical practice while maintaining sufficient methodological control over conventional treatment exposure.

Participants will also continue receiving their usual medical care, including any prescribed pharmacological treatments, which will not be modified as part of the study. Any relevant changes in medication during the study period will be recorded.

#### Music-based intervention

The intervention is defined as a structured music-based intervention rather than a formal music therapy intervention. This terminology was selected because the program will be delivered by a healthcare professional with formal musical training and experience in rehabilitation contexts, but not by a formally credentialed music therapist. Accordingly, the intervention is not presented as clinical music therapy and does not claim to replace treatment delivered by a qualified music therapist. Instead, it is conceived as a standardized, rehabilitation-oriented, music-based program designed to complement usual post-stroke rehabilitation care.

The intervention is based on a neuroscience-informed and integrative music-based approach, combining rhythm-based, instrumental, and structured musical activities to primarily support motor rehabilitation while incorporating cognitive and emotional components. Activities involving rhythmic sequencing, improvisation, and structured musical interaction are designed to engage executive functions, such as cognitive flexibility, working memory, and inhibitory control. Cognitive neuroscience has indicated that musical rhythm and structured auditory–motor activities recruit frontal and frontoparietal networks involved in executive functioning (10, 20, 21). These activities are included to increase cognitive engagement during motor and musical tasks rather than to constitute a stand-alone cognitive rehabilitation program.

The intervention is not intended to replicate standardized neurologic music therapy (NMT) protocols, but to adapt selected rhythm- and instrument-based principles to the aims and constraints of an outpatient post-stroke rehabilitation setting. The rhythmic components are informed by rhythmic auditory stimulation (RAS), particularly the use of an external, predictable beat to support movement timing, synchronization, and coordination (25). In this protocol, these principles are mainly adapted to seated upper-limb and whole-body coordination tasks, rather than to formal gait training. Instrument-based motor activities are conceptually related to therapeutic instrumental music performance (TIMP), which is defined in the updated second edition of the Handbook of Neurologic Music Therapy as a technique that uses structured musical instrument playing to facilitate upper-limb movement, coordination, and functional motor recovery in neurological populations (26). In the present intervention, TIMP-related principles are applied through goal-directed percussion and melodic-instrument tasks, with the instrument position, movement amplitude, rhythmic complexity, and level of cueing adapted to each participant’s motor abilities and fatigue.

The program was designed as an integrative and flexible intervention adapted to an outpatient rehabilitation setting, allowing adjustment of task complexity, level of motor engagement, and cognitive demands according to participants’ functional abilities and progression throughout the intervention. The intervention will be delivered by a nurse with experience in the management of stroke patients and formal professional musical training, with support from a professional experienced in post-stroke rehabilitation. Intervention facilitators will receive specific training in the study protocol before the start of the intervention at each participating center, including session structure, safety procedures, adaptation criteria, documentation of participant responses, and fidelity monitoring.

Previous studies on music-based interventions, neurologic music therapy, and music-supported rehabilitation in stroke populations report considerable heterogeneity in the intervention duration and intensity, with no clear consensus on the optimal dosage (13, 17). However, many studies conducted in chronic stroke populations have implemented structured multi-session interventions with frequencies ranging from one to three sessions per week over several weeks (22, 27), providing a general reference framework for intervention design. Accordingly, the intervention dose (10 sessions delivered twice weekly over 5 weeks, including 6 individual and 4 group sessions) was determined to be consistent with commonly used intervention ranges reported in previous music-based and neurologic rehabilitation studies (13, 17, 22, 27), while also considering feasibility within a real-world outpatient rehabilitation setting. This schedule was selected to balance intervention intensity, participant burden, and adherence while allowing evaluation of participant response.

The intervention has been described in accordance with recommendations for reporting music-based interventions, including the original Reporting Guidelines for Music-Based Interventions (RG-MBI) recommendations and the updated RG-MBI checklist (28, 29). The description specifies the theoretical rationale, intervention components, delivery format, intervention setting, musical materials and parameters, facilitator training, tailoring and progression rules, and treatment fidelity procedures.

This structured format enables standardized delivery while maintaining flexibility for individual adaptation.

Musical parameters, including tempo, rhythmic complexity, movement amplitude, instrument type, and level of facilitator cueing, will be adapted according to participants’ motor capacity, fatigue, comprehension, and emotional response. Tasks will begin at a level that allows successful participation and will progress by increasing rhythmic complexity, tempo, task duration, bilateral involvement, range of movement, memory load, or degree of independence. Task difficulty will be reduced if participants show excessive fatigue, frustration, loss of postural control, inability to follow instructions, or emotional discomfort. Treatment fidelity will be supported through facilitator training, use of the standardized session protocol, and completion of a session checklist documenting attendance, activities delivered, adaptations made, participant engagement, adverse events, and deviations from the protocol.

Session checklists will be reviewed periodically by the research team to monitor adherence to the protocol and identify deviations requiring corrective action.

The intervention consists of a 10-session structured program delivered twice per week over 5 weeks, including 6 individual sessions and 4 group-based sessions.

- Individual sessions (30–40 min): Focused on evaluation and individualized work on motor engagement, rhythmic participation, and emotional regulation.
- Group sessions (45 min): Conducted collectively with all participants, focused on social interaction, motor coordination, and emotional support.

These group-based and receptive components are informed by music therapy and music-based rehabilitation approaches commonly used to support emotional regulation, social interaction, and quality of life in rehabilitation populations. Group singing, shared rhythmic activities, and guided listening are consistent with rehabilitation-oriented music-based frameworks that emphasize emotional expression, social engagement, and patient-centered rehabilitation (6, 7).

#### Condensed session overview

The intervention follows a progressive structure combining individual and group-based sessions. A condensed overview is provided below, while the full session-by-session protocol, including detailed objectives, activities, facilitation procedures, and adaptation options, is provided in Supplementary material 1.

- Session 1 (Group, 45 min): Introduction to the program, establishment of group trust, vocal and physical warm-up with music, group percussion activities, and a closing group song to foster cohesion and shared participation.
- Session 2 (Individual, 30–40 min): Initial assessment of rhythmic motor participation, emotional response to music, vocal engagement, and ability to follow musical and verbal cues.
- Session 3 (Individual, 30–40 min): Initiation of individualized activities based on the initial assessment, including instrumental coordination tasks, breathing and relaxation exercises with music, and vocal rhythm/articulation exercises.
- Session 4 (Group, 45 min): Group rhythm and coordination activities, simple musical improvisation, and group singing to promote social interaction, participation, and vocal engagement.
- Sessions 5 and 6 (Individual, 30–40 min each): Progression of task complexity according to individual needs, including bilateral motor tasks, faster or more complex rhythmic patterns, relaxation techniques, and more demanding vocal-rhythmic exercises.
- Session 7 (Group, 45 min): Reinforcement of previous achievements through instrument practice, guided visualization with music, and reading or vocal engagement activities to support coordination, attention, and emotional wellbeing.
- Sessions 8 and 9 (Individual, 30–40 min each): Consolidation and refinement of acquired skills through review of individualized motor tasks, relaxation activities, and advanced vocal or rhythmic exercises.
- Session 10 (Group, 45 min): Integration and closure of the program through group rhythm dynamics, collaborative music creation, feedback, individualized recommendations, and a personally meaningful closing song selected by the participant.

The detailed protocol in Supplementary material 1 includes the specific objectives, activities, facilitation procedures, and adaptation options for each session.

Ten-session structured music-based intervention: session schedule (Table 1).

**Table 1:** Ten-session structured music-based intervention: session schedule.

Week	Session	Format	Duration	Main components	Objective
1	S1	Group	45 min	Welcome, warm-up with music, group percussion, and group song	Social interaction, trust-building, rhythmic engagement
1	S2	Individual	30–40 min	Rhythmic motor assessment, emotional listening, and singing/vocal rhythm	Motor engagement, emotional response, vocal participation
2	S3	Individual	30–40 min	Instrumental coordination, guided breathing, and rhythmic articulation	Coordination, emotional regulation, vocal participation
2	S4	Group	45 min	Group percussion, improvisation, and group song	Social interaction, rhythmic coordination, vocal participation
3	S5	Individual	30–40 min	Bilateral motor skills, faster tempos, and complex phrases	Fine motor skills, vocal participation
3	S6	Individual	30–40 min	Advanced relaxation and long-phrase singing	Fine motor skills, vocal participation
4	S7	Group	45 min	Instrument practice, visualization, reading, and vocal participation	Coordination, attention, emotional engagement
4	S8	Individual	30–40 min	Intensive motor review and deep relaxation	Motor consolidation
5	S9	Individual	30–40 min	Advanced vocal tasks and recitation/singing	Vocal-rhythmic control and motor consolidation
5	S10	Group	45 min	Group music creation, feedback, and meaningful song	Integration, motivation, emotional closure

### Follow-up

A follow-up interview will be scheduled 3 months after completion of the intervention to explore the maintenance of potential effects, the continued use of music-based strategies at home, and participants’ perceived acceptability, usefulness, and relevance of the intervention in their daily lives.

### Assessment

Assessments will be conducted at three main time points, plus one follow-up:

- Baseline (week 0): Prior to the start of the program. Includes assessment of motor, cognitive, and quality-of-life domains.
- Week 5 (post-intervention for the experimental group): Repetition of all measures to evaluate initial changes in the experimental group compared to the waitlist group.
- Week 10 (after the waitlist group intervention): Comprehensive evaluation of both groups following completion of the intervention.
- Three-month follow-up: Additional evaluation to explore maintenance of potential effects, including quality of life and daily functioning.

Standardized instruments will be used at all time points to measure motor, cognitive, and quality-of-life outcomes. In addition, participants’ perspectives on acceptability and perceived usefulness will be explored qualitatively during the 3-month follow-up interview, in line with multidomain rehabilitation research (12, 30). When feasible, caregivers or family members will also be invited to provide complementary perspectives on functional changes and everyday impact.

Outcome assessments will be conducted by professionals with a clinical background in neuropsychology or rehabilitation. All assessors will receive standardized training in the administration and scoring of the study measures before participant recruitment to ensure consistency and reliability of data collection.

Standard Protocol Items: Recommendations for Interventional Trials (SPIRIT) schedule of enrollment, interventions, and assessments (Table 2).

**Table 2:** Standard Protocol Items: Recommendations for Interventional Trials (SPIRIT) schedule of enrollment, interventions, and assessments.

Activity	-t1	Baseline (week 0)	Mid-intervention (week 3)	Post-phase 1 (week 5)	Post-crossover (week 10)	Follow-up (3 months)
Eligibility screening	X					
Informed consent		X				
Randomization		X				
Structured music-based intervention: experimental group		X	X	X		
Structured music-based intervention: control/waitlist group				X	X	
Adherence		X	X	X	X	
Assessment: FMA, Action Research Arm Test (ARAT), and WCST		X		X	X	X
Assessment: Behavior Rating Inventory of Executive Function–Adult Version (BRIEF-A)		X		X	X	X
Assessment: Stroke-Specific Quality of Life Scale (SS-QOL)		X		X	X	X
Adverse event reporting		X	X	X	X	X

X indicates that the corresponding activity, intervention, assessment, or reporting item takes place at that time point.

### Outcomes

#### Primary outcome

##### Fugl-Meyer Assessment (FMA)

The FMA is a stroke-specific, performance-based measure designed to assess motor impairment following stroke. It evaluates motor functioning, balance, sensation, and joint functioning, with particular emphasis on voluntary movement patterns. In this study, the motor domain score (including the upper and lower extremity motor subscales) will be used as the primary outcome variable. The motor domain is composed of ordinal items assessing movement, coordination, and reflex activity. The FMA has demonstrated excellent reliability, validity, and responsiveness in stroke populations and is widely considered one of the gold-standard measures for assessing post-stroke motor function in clinical and research settings (31, 32). It was selected as the primary outcome due to its sensitivity to changes in motor performance and its extensive use in stroke rehabilitation trials, allowing comparison with previous studies. It will constitute the main variable for the primary inferential analysis, while the ARAT will be analyzed as a secondary motor outcome, and executive functioning and quality-of-life measures will be interpreted as exploratory outcomes. Items are scored on a 3-point ordinal scale (0–2) and summed to yield a total motor score ranging from 0 to 100, with higher scores indicating better motor function (31, 32).

#### Secondary motor outcomes

##### Action Research Arm Test (ARAT)

The ARAT is a standardized, task-based observational measure of upper-limb function. It assesses grasp., grip, pinch, and gross movement through 19 items grouped into four subscales reflecting functional hand use. The total ARAT score will be used as the main variable for this measure. The ARAT has demonstrated strong reliability, validity, and responsiveness in stroke populations and is particularly sensitive to functional improvements in upper-limb activity (33).

It was selected to complement the FMA by providing an activity-level assessment of upper-limb function, thereby capturing clinically meaningful changes in functional performance. The total score ranges from 0 to 57, with higher scores indicating better upper-limb functional performance (33).

#### Exploratory outcomes

Executive functioning and stroke-specific quality of life will be examined as exploratory outcomes. This classification reflects the relatively brief duration of the intervention, consisting of 10 sessions over 5 weeks, and acknowledges that measurable changes in executive functioning and quality of life may require longer or more intensive interventions. These outcomes are included to explore potential broader effects of the intervention and to inform the design of future studies.

##### Wisconsin Card Sorting Test (WCST)

The WCST is a neuropsychological measure of executive functioning, particularly cognitive flexibility, set-shifting, and problem-solving ability. It requires participants to match stimulus cards according to changing sorting rules based on feedback, thereby assessing the ability to adapt to shifting task demands (34). Responses are evaluated across multiple indices, including categories completed and errors, with perseverative errors reflecting difficulty in cognitive flexibility. In this study, the number of perseverative errors will be used as an exploratory executive function variable, as it is considered one of the most sensitive indicators of cognitive inflexibility and impaired set-shifting. The WCST has been widely used in neurological populations, including stroke, and demonstrates good validity for assessing frontal lobe-related executive processes (34). It was selected due to its sensitivity to executive dysfunction commonly observed after stroke and its ability to capture changes in cognitive flexibility targeted by the intervention.

Perseverative errors are reported as a raw score, with higher values indicating greater executive dysfunction (34).

##### Behavior Rating Inventory of Executive Function–Adult Version (BRIEF-A)

The BRIEF-A is a standardized questionnaire designed to assess executive functioning in everyday life. It includes both a self-report version and an informant-report version completed by a caregiver or close relative, capturing ecologically valid aspects of executive behavior.

In this study, the Global Executive Composite (GEC) score will be used as an exploratory measure of everyday executive functioning. The GEC is derived from multiple subscales reflecting behavioral regulation and metacognitive processes. Both self-report and informant-report versions will be collected. The self-report version will be considered the main source for exploratory analysis, while the informant-report version will be used as complementary information. In cases of discrepancy between versions, the results will be reported descriptively and interpreted cautiously. The BRIEF-A has demonstrated good reliability and ecological validity in neurological populations. It is particularly useful for capturing executive dysfunction as it manifests in daily life, complementing performance-based measures such as the WCST (35). It was selected to provide an assessment of executive functioning in everyday life, reflecting real-world cognitive difficulties that may not be fully captured by laboratory-based tests. Scores are standardized T-scores (mean = 50, SD = 10), with higher scores indicating greater executive dysfunction (35).

##### Stroke-Specific Quality of Life Scale (SS-QOL)

The SS-QOL is a validated, stroke-specific self-report instrument designed to assess health-related quality of life across multiple domains relevant to stroke survivors. These domains include mobility, self-care, mood, energy, social roles, communication, cognition, and upper extremity function. In this study, the total SS-QOL score will be used as an exploratory measure of stroke-specific quality of life. The scale consists of multiple items rated on a Likert-type scale, with domain scores summed to obtain a total score reflecting the overall quality of life. The SS-QOL has demonstrated good reliability and validity in stroke populations and is widely used in rehabilitation research to assess patient-centered outcomes (36). It was selected due to its comprehensive and stroke-specific nature, allowing evaluation of the broader impact of the intervention on daily functioning, emotional wellbeing, and social participation. The total score ranges from 49 to 245, with higher scores indicating better quality of life (36).

#### Adherence and acceptability outcomes

Adherence, session attendance, and completion of the intervention will be documented descriptively. Participant-reported acceptability will be explored in the 3-month follow-up interview, focusing on perceived usefulness, burden, and relevance of the integrative format for everyday functioning.

When available, caregiver or family member perspectives will also be collected to complement participant-reported outcomes.

#### Safety outcomes

The intervention is considered low risk as it does not involve invasive or pharmacological procedures.

Adverse events will be systematically recorded in each session using a standardized form. Particular attention will be paid to potential adverse effects such as fatigue, frustration, emotional discomfort, or irritability. Adverse events will be reported to the ethics committee, and appropriate procedures will be followed for their management.

### Resources

The program requires the following resources:

- Physical space for sessions and assessments.
- Simple musical instruments: maracas, drums, xylophones, and tambourines.
- Audio equipment: speakers, music players, and microphones.
- Relaxation support materials: cushions and ergonomic chairs.

### Special considerations

- Individual adaptation: Activities will be tailored to the functional, cognitive, and emotional levels of each participant.
- Psychological follow-up: In cases where relevant emotional symptoms are detected, psychological support will be recommended.
- Family involvement: Family participation will be encouraged to provide continuous support and to foster a therapeutic environment at home.

### Data management

All data will be stored in a secure database accessible only to the authorized research team. Confidentiality will be ensured in accordance with current data protection regulations, and all personal information will be anonymized prior to analysis.

### Oversight and monitoring

The study will be coordinated by the principal research team, which will oversee recruitment, intervention, and data collection. Given the low-risk nature of this non-invasive, non-pharmacological intervention, an independent data monitoring committee will not be established.

### Dissemination policy

The results of the study will be disseminated through publications in scientific journals and presentations at specialized conferences. Participants requesting feedback will be provided with a summary of the study findings.

### Statistical analysis

Statistical analysis will be carried out using Jamovi (version 2.7.26). Qualitative variables will be described using frequencies (N) and percentages (%). Number of observations (N), mean, median, standard deviation, minimum, and maximum will be used to describe quantitative variables. Missing data will be reported descriptively, including the extent and pattern of missingness across time points.

Baseline demographic and clinical characteristics will be described. The outcome measures will be calculated according to their guidelines. Scores will be reported at all time points (baseline, 5 weeks, 10 weeks, and 3-month follow-up). A linear mixed-effects model (MLM) will be carried out to estimate the main effect of the intervention on the primary outcome, motor function assessed using the FMA. Additional models will be conducted to estimate the secondary motor outcome, upper-limb motor function assessed using the ARAT. Exploratory models will be conducted for cognitive flexibility (WCST), self-perceived executive functioning (BRIEF-A), and stroke-specific quality of life (SS-QOL). These exploratory analyses will be interpreted cautiously and will be used to inform future research rather than to establish definitive efficacy in these domains. The model will include time, group, and the interaction between group and time as fixed effects. Time after stroke will be included as a covariate to account for variability related to recovery stage. A random intercept will be specified to account for repeated measures. Model parameters will be estimated using restricted maximum likelihood (REML), which allows analysis without assuming sphericity. Degrees of freedom will be computed using the Satterthwaite method.

To explore agreement between participant and family member reports on the BRIEF-A executive function questionnaire, additional analyses will be carried out using intraclass correlation coefficients (ICC). Bland–Altman plots will be used to assess systematic bias and limits of agreement between informants.

### Dropout and missing data management

Given the longitudinal design of the study, particular attention will be paid to participant retention and data completeness. Protocol completion will be defined as participation in at least 80% of the scheduled sessions (i.e., at least 8 of 10 sessions).

The primary analysis will follow the intention-to-treat (ITT) principle, including all randomized participants with available outcome data in the analysis set. Linear mixed-effects models will use all available repeated-measures data under the missing-at-random assumption, without formal imputation. The extent and patterns of missing data will be reported descriptively.

An additional per-protocol analysis will be conducted, including only participants who meet the predefined completion threshold.

Dropout rates, reasons for withdrawal, and patterns of missing data will be reported descriptively to support transparent interpretation of the study findings.

## Discussion

While previous studies have reported benefits of music-based interventions, neurologic music therapy, and music-supported interventions in stroke rehabilitation, the majority of interventions have focused on isolated domains rather than adopting a multidimensional approach (8, 9, 21). Although contemporary rehabilitation frameworks emphasize the importance of integrating motor, cognitive, and psychosocial aspects of recovery (4, 5, 15), these domains are still often addressed separately in clinical practice, which may limit the overall effectiveness of rehabilitation programs.

Given the relatively brief duration of the intervention, the study is primarily powered and conceptually oriented toward motor outcomes. Executive functioning and stroke-specific quality of life are included as exploratory outcomes, as changes in these domains may require longer or more intensive interventions and may be influenced by multiple contextual and rehabilitation-related factors.

Therefore, the findings related to executive functioning and stroke-specific quality of life will be interpreted as preliminary signals of potential broader effects rather than as confirmatory evidence of efficacy.

In particular, any observed changes in executive functioning or quality of life should be interpreted in relation to participants’ baseline impairment, concurrent rehabilitation exposure, emotional status, and social participation, rather than attributed solely to the music-based intervention.

The added value of this protocol lies not only in the structural organization of sessions but also in the integration of motor rehabilitation activities with exploratory executive and quality-of-life-related components within a single coherent intervention. While conventional post-stroke rehabilitation and many music-based interventions tend to address these domains separately or sequentially, this protocol is designed to address these domains within the same structured rehabilitation-oriented context. By combining rhythmic motor training, cognitively demanding musical tasks, and group-based activities aimed at emotional and social engagement, the program reflects the complex and interdependent nature of post-stroke recovery and aims to enhance the ecological validity and functional relevance of rehabilitation.

The main strength of this study lies in its integrative approach, delivered in a real-world outpatient rehabilitation setting. The use of standardized and validated outcome measures across domains will provide relevant data to inform future research and rehabilitation practice.

Nonetheless, the study’s primary aim is to evaluate the effects of the intervention on motor outcomes and to provide preliminary information on its potential role in post-stroke rehabilitation. Although this study is powered to detect clinically significant differences in motor outcomes, it should be interpreted as an initial evaluation of an integrative intervention model.

If the intervention demonstrates favorable results, it could be incorporated as a complementary strategy within multidisciplinary post-stroke rehabilitation programs. Future research should aim to replicate these findings in larger and multicenter studies.

### Limitations

This randomized waitlist-controlled intervention study presents several inherent limitations. First, outcome assessors will not be blinded, which may introduce measurement bias despite the use of standardized procedures and trained personnel. Second, although this study begins in a single outpatient rehabilitation center, recruitment is planned to proceed in successive phases across additional centers in the Community of Madrid. This phased recruitment strategy may improve feasibility but may also introduce some variability between centers, which will be addressed through standardized intervention procedures, assessor training, fidelity monitoring, and statistical adjustment when appropriate. Third, the relatively short duration of the intervention may reduce the magnitude of observable effects.

In addition, although the sample size was determined through an *a priori* calculation, potential dropout, variability in clinical profiles, and differences in recruitment flow across centers may still affect statistical power and study implementation. These factors should be considered when interpreting the findings. Future studies may explore longer or more intensive protocols and broader multicenter recruitment strategies to further evaluate the impact of integrative music-based interventions.

### Study status

Recruitment has not yet started. The first recruitment phase is planned to begin in a single outpatient neurological rehabilitation center in the Community of Madrid, with subsequent phases extending to additional centers until the required total sample size is achieved. Recruitment is expected to be completed within approximately 1 year. The study has been preregistered in OSF Registries. The protocol version is 1.0 (2 March 2026).

### SPIRIT guidelines

This protocol has been structured using the Standard Protocol Items: Recommendations for Interventional Trials (SPIRIT) statement as a reporting framework to ensure transparency and methodological quality. The schedule of enrollment, interventions, and assessments is presented in Table 2 following SPIRIT recommendations. The SPIRIT checklist is provided in the Supplementary material, following the SPIRIT 2025 statement (37).

In addition, the checklist itself has been completed and included in Supplementary material 2, with page references for each SPIRIT item.

## Footnotes

1
https://osf.io/hqruw
