# Supplementary material for: A structured music-based intervention for motor rehabilitation and exploratory cognitive and quality-of-life outcomes after stroke: a study protocol for a randomized waitlist-controlled intervention study
Source: Front Neurol. 2026 Jul 1;17:1822193. doi: 10.3389/fneur.2026.1822193 (PMC13368994; doi:10.3389/fneur.2026.1822193)
Supplement: Supplementary file 1 [file Supplementary_file_1.DOCX]

**Session descriptions - Music therapy as a tool for psychomotor**

**Session descriptions**

**Session 1 (Group, 45 minutes):**

- Objectives: Introduction, establishing a climate of trust, and fostering interaction.
- Activities:
  - Introduction and welcome: The therapist and participants briefly introduce themselves to create a warm and trusting atmosphere. This may include mentioning participants’ names and providing a short explanation of what to expect during the session.
  - Vocal and physical warm-up with music: Breathing exercises and gentle stretches performed while listening to calm instrumental music. These exercises help relax the body and prepare the mind for the session.
  - Group rhythm exercises using percussion instruments: Participants use maracas, tambourines, or drums to perform rhythmic exercises. They follow a simple beat first individually and then collectively, aimed at improving coordination and group synchronization.
  - Closing group song: A simple song is chosen for everyone to sing together. This activity seeks to strengthen group cohesion and provide a shared experience.

**Session 2: Individual (30–40 minutes)**

- Objectives: Assess individual motor, emotional, and communication capacities.
- Activities:
  - Rhythmic mobility assessment: The patient performs simple movements (e.g., clapping, tapping feet) in time with a steady rhythm. Amplitude, fluency, and coordination of movements are evaluated.
  - Emotional response to music: The patient listens to different types of music (calm, joyful, melancholic), while the therapist observes emotional responses such as facial expressions, body language, and verbal comments.
  - Singing and speech exercises: The patient sings short phrases or repeats words and sentences to music. Clarity of speech, intonation, and ability to follow rhythm are assessed.

**Session 3: Individual (30–40 minutes)**

- Objectives: Initiate personalized interventions based on the initial evaluation.
- Activities:
  - Motor coordination with instruments: The patient plays simple rhythmic patterns on instruments such as xylophones or small drums, gradually increasing complexity according to progress.
  - Breathing and relaxation techniques with music: Controlled breathing exercises synchronized with slow music to reduce anxiety and improve lung capacity.
  - Pronunciation and rhythm exercises: The patient repeats words or phrases to music, focusing on clarity of articulation and synchronization with rhythm.

**Session 4: Group (45 minutes)**

- Objectives: Strengthen group work and social interaction.
- Activities:
  - Group rhythm and coordination: Collective percussion exercises in which participants coordinate to create a shared rhythm.
  - Musical improvisation: Use of simple instruments or voice to improvise melodies and rhythms, encouraging creativity, self-expression, and confidence within the group.
  - Group song for communication: Singing a song with a simple refrain that allows everyone to participate, reinforcing communication, memory, and language.

**Sessions 5 and 6: Individual (30–40 minutes each)**

- Objectives: Intensify therapy according to specific needs.
- Activities:
  - More complex motor tasks: Exercises requiring bilateral movements (e.g., playing with both hands) or faster rhythms, adapted to the patient’s recovery level.
  - Advanced relaxation techniques: Visualization of calming scenes while listening to music to reduce stress and improve focus.
  - Language exercises with greater difficulty: Longer phrases or words with complex sounds to improve articulation and speech fluency.

**Session 7: Group (45 minutes)**

- Objectives: Reinforce achievements and address new areas for improvement.
- Activities:
  - Instrument practice for coordination: More complex pieces or rhythmic sequences are practiced to enhance coordination.
  - Guided visualization with music: Patients imagine positive scenarios while listening to calm or joyful music, reinforcing emotional well-being.
  - Language and communication exercises: Reading short texts or song lyrics aloud, followed by singing, to improve reading comprehension and verbal fluency.

**Sessions 8 and 9: Individual (30–40 minutes each)**

- Objectives: Refine acquired skills and prepare for program closure.
- Activities:
  - Intensive review of motor skills: Repetition of the most effective motor exercises from the program to consolidate progress and encourage continued practice.
  - Final relaxation session: Deep relaxation techniques with music to end with a sense of well-being.
  - Advanced speech and communication tasks: Recitation of short poems or singing tasks requiring greater articulation and vocal control.

**Session 10: Group (45 minutes)**

- Objectives: Consolidate group work and evaluate collective progress.
- Activities:
  - Group rhythm dynamics: Activities where participants work together to maintain a steady rhythm, such as each adding a sound in a circle.
  - Collaborative music creation: The group collectively creates an improvised musical piece, contributing melodies, rhythms, or lyrics, to foster creativity and collaboration.
  - Closing group feedback: Participants share their experiences, while the therapist provides constructive and positive feedback, highlighting individual and group achievements.
  - Final clinical review with individualized recommendations: Summary of progress and personalized advice for continued recovery, including suggested musical activities to perform independently or with family support.
  - Clinically meaningful closing song chosen by the patient: The session ends with a song identified by the patient as personally significant, to encourage emotional closure and motivation.
